# Microvillus Inclusion Disease Caused by *MYO5B*: Different Presentation and Phenotypes Despite Same Mutation

**DOI:** 10.1097/PG9.0000000000000309

**Published:** 2023-05-09

**Authors:** Bente Utoft Andreassen, Lise Aunsholt, Elsebet Østergaard, Jakob Ek, Lisa Leth Maroun, Marianne Hørby Jørgensen

**Affiliations:** From the * Department for Children and Adolescent, Rigshospitalet, Copenhagen University Hospital, Denmark; † Department of Neonatology, Rigshospitalet, Copenhagen University Hospital, Denmark; ‡ Department of Genetics, Rigshospitalet, Copenhagen University Hospital, Denmark; § Department of Clinical Medicine, University of Copenhagen, Copenhagen, Denmark; ∥ Department of Pathology, Rigshospitalet, Copenhagen University Hospital, Denmark.

**Keywords:** MYO5B, cholestasis, malabsorption, childhood

## Abstract

Microvillus inclusion disease (MVID) is associated with specific variants in the *MYO5B* gene causing disrupt epithelial cell polarity. MVID may present at birth with intestinal symptoms or with extraintestinal symptoms later in childhood. We present 3 patients, of whom 2 are siblings, with *MYO5B* variants and different clinical manifestations, ranging from isolated intestinal disease to intestinal disease combined with cholestatic liver disease, predominant cholestatic liver disease clinically similar to low-gamma-glutamyl transferase PFIC, seizures, and fractures. We identified 1 previously unreported MYO5B variant and 2 known pathogenic variants and discuss genotype–phenotype correlations of these variants. We conclude that MVID may present phenotypically different and mimic other severe diseases. We suggest that genetic testing is included early during diagnostic investigations of children with gastrointestinal and cholestatic presentation.

Microvillus inclusion disease (MVID) is mainly caused by pathogenic variants in *MYO5B* gene. MVID may present as a severe congenital enteropathy with intractable watery diarrhea, most often with onset immediately after birth, or present extraintestinal with persistent, low-gamma-glutamyl transferase cholestatic liver disease with increased concentrations of primary bile acids and progressive liver damage. Hence, the latter presentation is clinically indistinguishable from progressive familial intrahepatic cholestasis (PFIC) types I, II, IV, and V (1–3). More than a hundred *MYO5B* pathogenic variants have been reported (4–7). A complete lack of *MYO5B* protein causes predominant intestinal disease (MVID), whereas the expression of full-length mutant *MYO5B* protein with residual function causes predominant cholestatic liver disease (MVID-PFIC), and the expression of mutant *MYO5B* proteins without residual function causes both intestinal and hepatic dysfunction (MVID-mixed) (6,8).

The diagnosis of MVID is established with a combination of molecular genetic testing, light microscopic detection of vacuoles in the enterocytes, and ultimately, electron microscopic detection of microvillus inclusions. Enterocyte-to-enterocyte variation exists, and the percentage of enterocytes with microvillus inclusions varies greatly between patients and in a single patient. Liver biopsy shows portal and lobular fibrosis and giant cell transformation (5).

In this article, we present 3 cases with *MYO5B* variants, of which 1 is not previously published, and discuss genotype/phenotype correlations associated with the variants.

## CASES

### Patient 1

This patient was a girl born at gestational age 36 weeks + 4 days, with a birth weight of (BW) 3600 g. She was breastfed. When she was 7 days old, she was hospitalized due to a loss of 17% of her weight, lethargy, and multiple large stools. Blood samples showed hypoglycemia, metabolic acidosis with normal lactate and low bicarbonate (Table 1). Enteral feeding exacerbated the metabolic acidosis and stool output. Metabolic screening of urine was normal and showed no renal loss of bicarbonate. She received total parenteral nutrition and gained weight satisfyingly. Whole genome sequencing was performed at age 14 days showing that she was compound heterozygous for 2 *MYO5B* variants, (Table 2), of which 1 was novel. These findings were consistent with early onset MVID, which was confirmed by histologic and electron microscopic (EM) examination of small intestinal biopsies. She died at 7 weeks of age.

**TABLE 1.** Initial presentation of the children with *MYO5B* variants

Child	1	2	3
Sex	f	m	f
Age at onset, months	**0**	**5**	**3**
Ethnicity	Danish	Middle East	Middle East
Diarrhea	Yes	No	No
ALT/ALP at admission (ref. 8–32/17–46) U/L	41/368	139/196	94
GGT (ref. 10–45) U/L	-	56	15
Bilirubin total/conjugated (ref. 5–25/<17) µmol/L	197/48	85/66	63/-
INR (ref. <1.3)	0.7	1.1	10
Fractures	No	Yes	No

Child 1 died 7 week of age of septicemia; Child 2 and 3 are siblings.

ALT = alanine aminotransferase; ALP = alkaline phosphatase; GGT = gamma-glutamyl transferase; INR = International Normalized Ratio.

**TABLE 2.** *MYO5B* variants in the 3 children

Case		Variant	Amino acid change	Predicted effect	Reference (5,9)
1	Compound heterozygous	c.655C>T c.1677C>G	p.(Arg219Cys) p.(Tyr559*)	Missense Nonsense	PMID: 33525641 Novel
2	Homozygous	c.244G>A	p.(Glu82Lys)	Missense	PMID: 28407399
3	Homozygous	c.244G>A	p.(Glu82Lys)	Missense	PMID: 28407399

### Patient 2

This patient was a boy, born at term to first cousin parents. His BW was 3820 g, and he was breastfed. He presented with prolonged, conjugated hyperbilirubinemia at 5 months of age and had no gastrointestinal symptoms. Diagnostic work-up for cholestatic liver disease was inconclusive and he was discharged. At the ages of 15 and 18 months, he had 2 separate fractures in the upper and lower extremities, respectively (battered child was first suspected). Blood samples indicated rickets and he was treated with vitamin D, calcium, and phosphate.

He was admitted to hospital several times in the first years of life with passing diarrhea and vomiting, and a diagnosis of chronic cholestasis was established when he was 2.9 years old. Whole exome sequencing (WES) was performed at 5 years of age and showed a previously reported homozygous variant in *MYO5B* (Table 2). Intestinal histology and EM were consistent with MVID.

### Patient 3

The younger sister of patient 2 was born at term with a BW of 3470 g and was breastfed. During her first year of life, she had episodes with diarrhea and vomiting. At 9 months of age, just 1 month after her older brother’s chronic cholestasis was diagnosed, she was admitted to hospital because of nonfebrile seizure, and she was diagnosed with intracerebral hemorrhage, caused by high prothrombin time with an International Normalized Ratio of 10 (<1.3). The International Normalized Ratio normalized after intravenous vitamin K administration. Blood samples showed mild conjugated hyperbilirubinemia and mild liver transaminase elevation, and a liver biopsy showed progressive familiar intrahepatic cholestasis, with no fibrosis or giant cells. At 3 years of age, WES was performed showing the same homozygous *MYO5B* variant as in her brother (child 2). Intestinal histology and EM revealed subtle changes that could be compatible with MVID.

**FIGURE 1.**
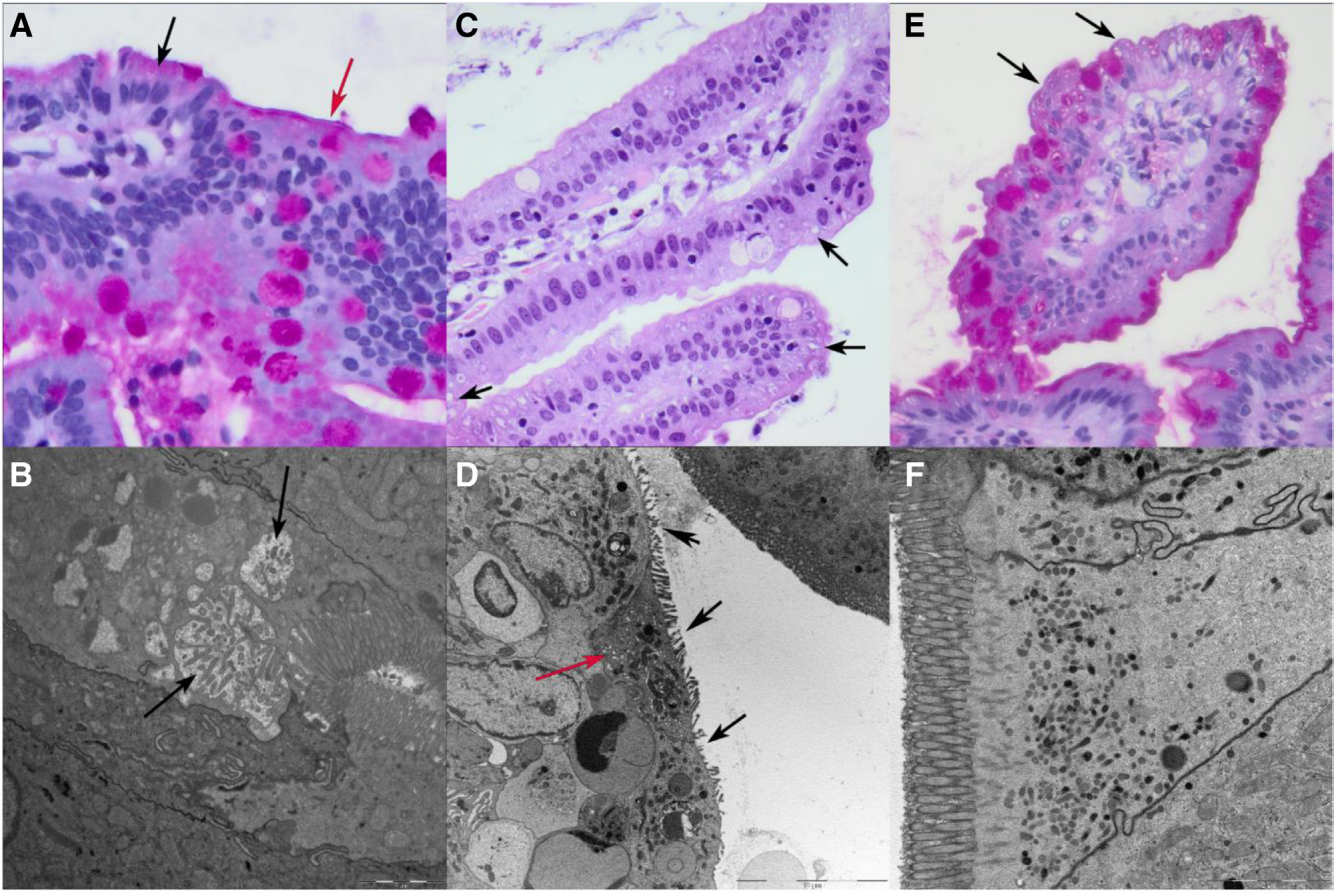
Light (top row) and electron microscopy (bottom row, fixative glutaraldehyde) of duodenal biopsies from patients 1 to 3 (left to right column). A) Villous top with PAS-positive subapical material and vacuoles (black arrow) and double contour of the brush border (red arrow) (PAS stain, ×600). B) Epithelial cell with microvillus inclusions (black arrows). C) Villi with disrupted brush border and cytoplasmic vacuoles (black arrows) (hematoxylin and eosin stain, ×400). D) Segmental shortening/loss of microvilli (black arrows) and subapical tubulo-vesicular structures (red arrow). E) Villous top with subapical vacuoles (black arrows) (hematoxylin and eosin stain, ×400). F) Epithelial cells with no specific findings.

The clinical and molecular genetic data are summarized in Tables 1 and 2, and a comparison of the clinical findings in patient 2 and 3 are shown in Table 3 and figure 1.

**TABLE 3.** Comparison of current status of siblings with a homozygous *MYO5B* variant

	Case 2 (12-year-old boy)	Case 3 (10-year-old girl)
INR elevation	No	No
Episodes of diarrhea necessitating hospital admission	No	Yes
Treatment with fat-soluble vitamin supplements	Yes	Yes
Height *z* score/weight *z* score	−0.45/−1.01	−2.62/−2.81
Bone age, y	11.81	7.49
Bone mass density *z* score	−0.8	−2.1
Severe cholestasis	Yes	Yes
ALT/ALP (ref. 8–32/17–46) U/L	21/990	41/694
Bile salt (ref. <17) µmol/L	>150	26
Itching	Yes	No
Number of anticholestatic medications	4	2

Cholestatic medication: Ursodeoxycholic acid, Rifabutin, Cholestyramine, Naltrexone.

ALT = alanine aminotransferase; ALP = alkaline phosphatase; INR = International Normalized Ratio.

## DISCUSSION

Here we present 3 patients with *MYO5B* variants and different clinical manifestations, ranging from isolated intestinal disease to intestinal disease combined with cholestatic liver disease, predominant cholestatic liver disease clinically similar to low-gamma-glutamyl transferase PFIC, seizures, and fractures.

The homozygous variant found in the 2 siblings (p.Glu82Lys) has previously been reported homozygous in 1 patient with MVID presenting at age 6 months (7) and later development of PFIC, thus a mixed phenotype. It was also reported in a pair of siblings with onset of MVID within the first months and first year of life, respectively, which later resolved, and the patients have no sign of liver affection (10). The 2 siblings reported here belong to 2 different phenotype categories, that is, PFIC for patient 2 who had no clinical symptoms of MVID, and the mixed phenotype for patient 3 (Tables 1 and 3). Thus, for this specific variant, it is not possible to predict phenotype from genotype, because it has now been found homozygous in both the patients with PFIC, MVID, and MVID-PFIC. To our knowledge, this is the only *MYO5B* variant that may be associated with all 3 phenotypes.

Patient 1 had severe MVID with early death. She harbored a novel nonsense variant (leading to p.[Tyr559*]) and a missense variant (p.Arg219Cys) that has been reported, but without any phenotype information. Another amino acid change at the same position (p.Arg219His) was reported in a patient with MVID who harbored another missense variant on the other allele (5). The combination of a nonsense variant and (p.Arg219Cys) in a patient with a severe phenotype as found here could indicate that p.Arg219Cys is not associated with any residual function.

Possible genotype–phenotype correlation in *MYO5B*-associated disease has been addressed in a few studies (5,8). In the study by Wang et al (8) of 130 patients with *MYO5B* variants, they found that the severity of intestinal manifestation was positively correlated to the number of null variants. Children with cholestasis carried at least 1 non-null variant. Comparing MVID and combined patients to FIC patients, the latter were more likely to carry missense/in-frame variants affecting the nonmotor regions of MYO5B. In line with these results, Aldrian et al (5) studied 114 cases and found that biallelic truncating variants were associated with a phenotype of MVID or mixed MVID/PFIC, but not isolated PFIC, and concluded that MVID results from the lack of *MYO5B* in enterocytes causing disrupted enterocyte localization. Solitary primary cholestatic liver disease may result from the expression of mutant MYO5B proteins that cause aberrant protein–protein interactions in hepatocytes, while nonfunctional MYO5B protein causes both intestinal and hepatic disease. However, as the siblings we report here illustrate, care should be taken when drawing firm conclusions on predicted phenotype based solely on the identified variant(s), because the variant they carry is associated with all 3 phenotypes, indicating that other factors modify the phenotype.

We think the use of whole genome sequencing/WES is a valuable diagnostic tool for cholestatic liver disease, and we suggest that genetic testing is included early during diagnostic investigations of children with gastrointestinal and cholestatic presentation.

## ACKNOWLEDGMENT

Informed patient consent was obtained from all patients for publication of the case details.
